# Histological evaluation of acute ischemic stroke thrombi may indicate the occurrence of vessel wall injury during mechanical thrombectomy

**DOI:** 10.1136/neurintsurg-2021-017310

**Published:** 2021-05-11

**Authors:** Oana Madalina Mereuta, Mehdi Abbasi, Seán Fitzgerald, Daying Dai, Ram Kadirvel, Ricardo A Hanel, Albert J Yoo, Mohammed A Almekhlafi, Kennith F Layton, Josser E Delgado Almandoz, Peter Kvamme, Vitor Mendes Pereira, Babak S Jahromi, Raul G Nogueira, Matthew J Gounis, Biraj Patel, Amin Aghaebrahim, Eric Sauvageau, Parita Bhuva, Jazba Soomro, Andrew M Demchuk, Ike C Thacker, Yasha Kayan, Alexander Copelan, Pouya Nazari, Donald Robert Cantrell, Diogo C Haussen, Alhamza R Al-Bayati, Mahmoud Mohammaden, Leonardo Pisani, Gabriel Martins Rodrigues, Ajit S Puri, John Entwistle, Alexander Meves, Jorge L Arturo Larco, Luis Savastano, Harry J Cloft, David F Kallmes, Karen M Doyle, Waleed Brinjikji

**Affiliations:** 1 Department of Radiology, Mayo Clinic, Rochester, Minnesota, USA; 2 CÚRAM – SFI Research Centre for Medical Devices and Department of Physiology, National University of Ireland Galway, Galway, Ireland; 3 Department of Neurosurgery, Baptist Medical Center, Jacksonville, Florida, USA; 4 Department of Neurointervention, Texas Stroke Institute, Dallas-Fort Worth, Texas, USA; 5 Departments of Clinical Neurosciences, Radiology, and Community Health Sciences, Hotchkiss Brain Institute and Cumming School of Medicine, University of Calgary, Calgary, Alberta, Canada; 6 Department of Radiology, Baylor University Medical Center, Dallas, Texas, USA; 7 Department of NeuroInterventional Radiology, Abbott Northwestern Hospital, Minneapolis, Minnesota, USA; 8 Department of Radiology, University of Tennessee Medical Center, Knoxville, Tennessee, USA; 9 Departments of Medical Imaging and Surgery, Toronto Western Hospital, Toronto, Ontario, Canada; 10 Departments of Radiology and Neurosurgery, Northwestern University, Chicago, Illinois, USA; 11 Department of Neurology, Grady Memorial Hospital, Atlanta, Georgia, USA; 12 Emory University, Atlanta, Georgia, USA; 13 Department of Radiology, University of Massachusetts Medical School, New England Center for Stroke Research, Worcester, Massachusetts, USA; 14 Departments of Radiology and Neurosurgery, Carilion Clinic, Roanoke, Virginia, USA; 15 Department of Dermatology, Mayo Clinic, Rochester, Minnesota, USA; 16 Department of Neurosurgery, Mayo Clinic, Rochester, Minnesota, USA

**Keywords:** thrombectomy, stroke, vessel wall

## Abstract

**Background:**

Several animal studies have demonstrated that mechanical thrombectomy (MT) for acute ischemic stroke (AIS) may cause vessel wall injury (VWI). However, the histological changes in human cerebral arteries following MT are difficult to determine.

**Objective:**

To investigate the occurrence of VWI during MT by histological and immunohistochemical evaluation of AIS clots.

**Methods:**

As part of the multicenter STRIP registry, 277 clots from 237 patients were analyzed using Martius Scarlett Blue stain and immunohistochemistry for CD34 (endothelial cells) and smooth muscle actin (smooth muscle cells).

**Results:**

MT devices used were aspiration catheters (100 cases), stentriever (101 cases), and both (36 cases). VWI was found in 33/277 clots (12%). There was no significant correlation between VWI and MT device. The degree of damage varied from grade I (mild intimal damage, 24 clots), to grade II (relevant intimal and subintimal damage, 3 clots), and III (severe injury, 6 clots). VWI clots contained significantly more erythrocytes (p=0.006*) and less platelets/other (p=0.005*) than non-VWI clots suggesting soft thrombus material.

Thrombolysis correlated with a lower rate of VWI (p=0.04*). VWI cases showed a significantly higher number of passes (2 [1–4] vs 1 [1–3], p=0.028*) and poorer recanalization outcome (p=0.01*) than cases without VWI.

**Conclusions:**

Histological markers of VWI were present in 12% of AIS thrombi, suggesting that VWI might be related to MT. VWI was associated with soft thrombus consistency, higher number of passes and poorer revascularization outcome. There was no significant correlation between VWI and MT device.

## Introduction

Mechanical thrombectomy (MT) for acute ischemic stroke (AIS) has a risk of arterial wall damage that may lead to long-term side effects, such as intima hyperplasia, de novo stenosis, and occlusions and arterial dissection.^1–3^ The injury may occur in relation to the forces exerted by the device against the vessel wall,^1^ the occurrence of vasospasm,^2^ vascular anatomy,^3^ and the presence of underlying stenotic conditions, such as atherosclerotic plaque^4 5^ or calcification of the vessel wall.^6^

Several animal studies have evaluated the histological and angiographic changes in the vessel wall due to potential damage during MT but the results have been inconsistent.^1 5 7–11^ Initial studies found that Trevo and Solitaire devices can cause severe disruption of the intima^8 9^ and vascular damage extending to the medial layer.^10^ Another study that compared an aspiration device (Penumbra) with stentrievers (Merci, Catch, and Solitaire) demonstrated that all devices caused endothelial denudation and disruption of the internal elastic lamina, but the aspiration device was associated with more intimal and medial layer edema.^11^ However, a recent histological and ultrastructural study comparing direct aspiration first pass technique with stentrievers (Solitaire) showed that stentrievers can be more harmful to all layers of the vessel wall—in particular, the endothelium.^1^

Moreover, based on angiographic follow-up, Kurre *et al* 2 observed a decrease in arterial diameter at the site at which the thrombectomy was performed. A further study reported the appearance of a concentric arterial wall thickening and enhancement after MT using high-resolution contrast-enhanced vessel wall MRI.^12^

However, little is known about the extent and mechanism of vessel wall injury (VWI) caused by MT in human cerebral arteries. Therefore, the aim of our study was to investigate the presence of histological markers that may be indicative of VWI during MT by histological and immunohistochemical evaluation of endothelial cells, smooth muscle cells, and subendothelial connective tissue in AIS clots.

## Materials and methods

### Clot collection

This study was performed as part of the multi-institutional Stroke Thromboembolism Registry of Imaging and Pathology (STRIP). The study was approved by the institutional review board and was compliant with the Health Insurance Portability and Accountability Act. A waiver of consent was granted. Patients were included in the study if they were >18 years, had undergone mechanical thrombectomy treatment for AIS, and had embolic material available for analysis.

Data regarding suspected stroke etiology, occlusion location, and clot length on non-contrast computed tomography prior to MT, approach used for MT, number of procedural passes required to remove the clot, and final modified Thrombolysis in Cerebral Infarction (mTICI) score were self-reported at each center and captured on the STRIP registry data abstraction form. Stroke etiology was classified using the Trial of Org 10172 in Acute Stroke Treatment (TOAST) system: large artery atherosclerosis, cardioembolic stroke of other determined etiology, and cryptogenic.^13^

### Histological analysis of thrombi and extracted clot area measurement

**Figure 1:**
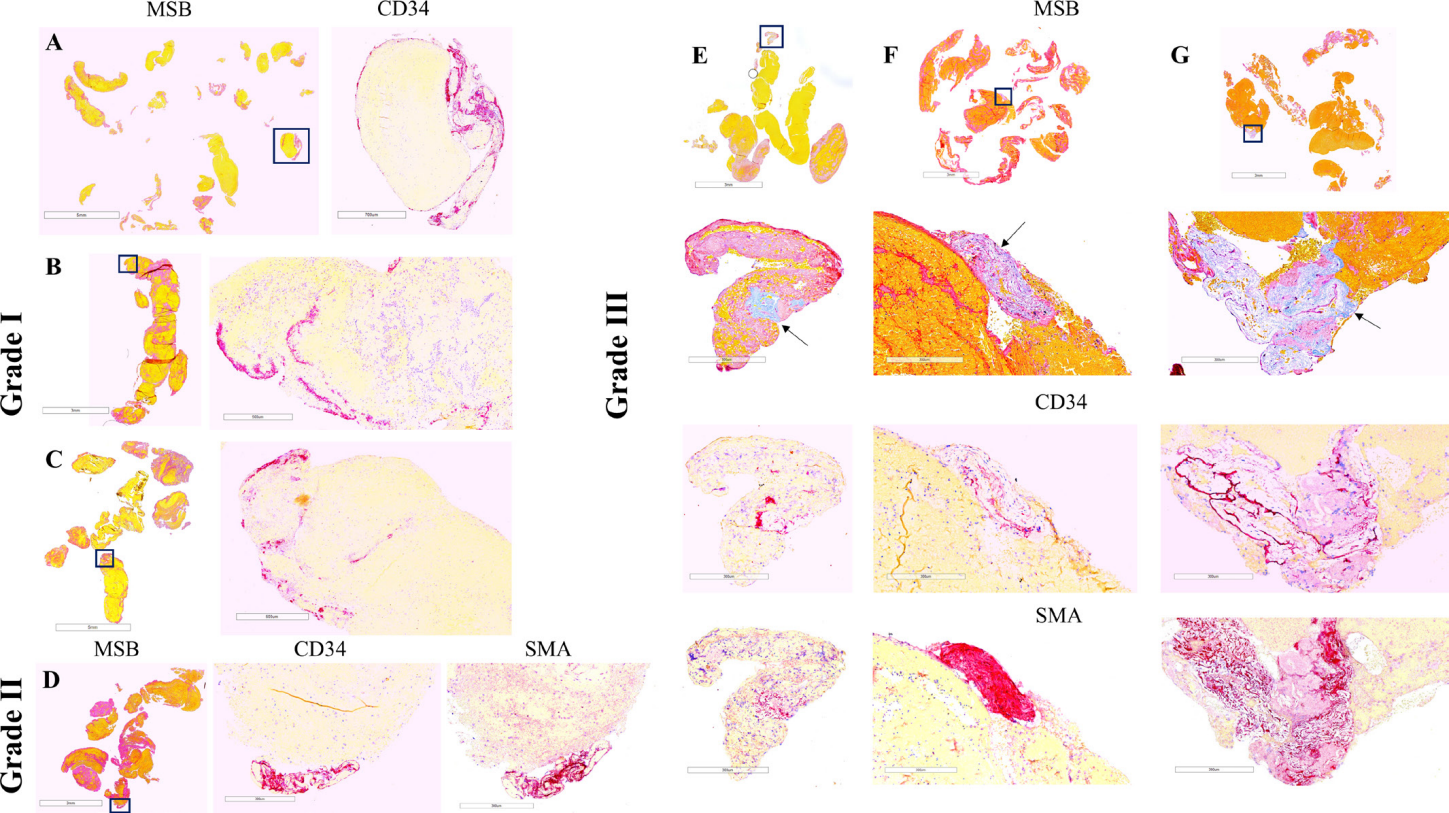
Thrombus histology and immunohistochemistry in representative cases selected by the degree of vessel wall injury and mechanical thrombectomy approach. Grade I: aspiration catheter (A), stentriever (B), and combination of devices (C); grade II: stentriever (D); grade III: aspiration catheter (E), stentriever (F), and combination (G). Martius Scarlett Blue (MSB) staining identifies the standard components of clots: red blood cells (yellow), fibrin (red). and platelets/other (light pink). Collagen can be also identified by MSB (E–G: light blue and arrows). Immunostaining for CD34 and smooth muscle actin (SMA) identifies endothelial cells and smooth muscle cells (purple), respectively. Areas within the squares in the MSB images are magnified in the immunostaining images. Note that clustered endothelial cells are distributed along the edge of clot fragments in grade I injury (A–C) while defined clusters of CD34- and SMA-positive cells are located at the periphery of the clot in grade II (D) and are associated with collagen in grade III (E–G). Scale bar (MSB)=5 mm (A, C), 3 mm (B, D), 3 mm and 300 µm (E–G); scale bar (immunostaining)=700 µm (A), 500 µm (B), 600 µm (C), and 300 µm (D–G).

On retrieval, each clot was immediately fixed in 10% phosphate-buffered formalin. Clots were shipped to the histology core facility. On arrival at the core facility, gross photos were taken of each clot. All clots were then processed using a standard tissue processing protocol, embedded in paraffin and cut into 3 µm sections. Two representative sections for each clot were stained with Martius Scarlett Blue (MSB) to identify the main components of clots (red blood cells, white blood cells, fibrin, platelets/other) as shown in figure 1.^14 15^

Stained slides underwent whole slide scanning (Motic Easyscan Pro, Motic Digital Pathology) at ×20 magnification. MSB quantification of clot components was performed using Orbit Image Analysis software (www.orbit.bio).^16 17^ ImageJ software (https://imagej.nih.gov/ij/) was utilized to measure total extracted clot area (ECA) of each clot using thegross image of the clot. We used the equations proposed by Duffy et al to calculate the overall contribution of each component to whole thrombus material.^18^

### Immunohistochemical analysis of thrombi

Immunostaining was used to identify endothelial cells (monoclonal mouse anti-human CD34, clone QBEnd-10, Dako M7165), smooth muscle cells (monoclonal mouse anti-human smooth muscle actin, SMA, clone 1A4, Dako M0851), and von Willebrand Factor (monoclonal mouse anti-human vWF, Dako M0616). Positive controls (artery tissue and human tonsil) and negative controls (omitting primary antibodies) were used. Immunohistochemistry was performed on a Leica Bond Max autostainer using a RedMap kit (Bond Polymer Refine Red Detection, Leica Biosystems).

The grading system showing the degree of underlying intimal damage associated with thrombi retrieved by MT was previously established and published by a board-certified neuropathologist.[19] The grading system used in our study was slightly modified by adding SMA immunohistochemistry to identify smooth muscle cells, another component of vessel wall.

The slides were examined by an experienced pathologist blinded to clinical data, and a grading system to classify the vessel wall damage was established (figure 1): grade I – clustered endothelial cells (CD34-positive cells) at the periphery of the clot: mild intimal damage; grade II – clustered endothelial cells associated with smooth muscle cells (SMA-positive cells) at the periphery of the clot: relevant intimal and subintimal damage; grade III - clustered endothelial cells and/or smooth muscle cells associated with subendothelial connective tissue (collagen) at the margin of the clot: severe injury of the vessel wall. The presence of single endothelial cells or small clusters of endothelial cells was probably related to the physiological turnover of the endothelium.^19^

### Statistical analysis

Data were analyzed using IBM SPSS 25 software. Graphpad Prism 8 was used to generate graphs. A Kolmogorov-Smirnov test indicated that quantitative variables did not follow a standard normal distribution and therefore, the non-parametric Kruskal-Wallis test was applied to make comparisons. Correlations between categorical variables were assessed using χ^2^ test. A level of statistical significance for all analyses was set at p<0.05 (two-sided). Results were reported as mean±SD, median (IQ1–IQ3), or number (% of cases), as appropriate.

## Results

### Clinical data

Two-hundred and thirty seven patients who underwent MT between October 2016 and November 2019 for large vessel occlusion in the anterior or posterior cerebral circulation were included in the study. Two hundred and seventy-seven clots were retrieved. Aspiration was used in 100 cases, stentriever in 101 cases, and a combined approach of stentriever and aspiration device was used in 36 cases. Suspected stroke etiologies were large artery atherosclerosis (43 cases, 18.1%), cardioembolic (100 cases, 42.2%), other determined etiology (19 cases, 8%), and cryptogenic (58 cases, 24.5%). The etiology was not reported in 17 cases (7.2%). Eighty patients (33.8%) were treated with intravenous recombinant tissue plasminogen activator (IV rtPA). Two hundred and seventeen patients (91.6%) had an occlusion in the anterior circulation, 16 cases (6.8%) in the posterior circulation, and one case (0.4%) had a dual occlusion involving both territories. The clots were extracted at first attempt in 130 cases (54.9%). Multiple procedural passes were required in 107 cases (45.1%). A final mTICI score 2b/3 was achieved in 214 patients (90.3%).

### Histopathologic findings

The overall contribution of each component based on the quantification of MSB staining was 52.16 ± 16.88% red blood cells (RBCs), 29.15 ± 12.83% fibrin, 15.17 ± 14.6% platelets/other and 3.48 ± 2.57% white blood cells for the VWI clots. Moreover, VWI clots contained significantly more RBCs (52.16 ± 16.88% vs 41.78 ± 21.32%, p=0.006*) and less platelets/other (15.17 ± 14.6% vs 23.12 ± 17.88%, p=0.005*) than non-VWI clots suggesting soft thrombus material. Total ECA was not significantly different between VWI and non-VWI group (43.17 ± 36.28 mm^2^vs 62.75 ± 82.52 mm^2^, p=0.233). Data are summarized in Supplementary Table 1.

### Degree of vessel wall damage

We found evidence of VWI in 33 clots (12%). The degree of damage was categorized as follows: grade I – 24 clots (72.7%), grade II – three clots (9.1%), and grade III – six clots (18.2%).

Grade I damage was the predominant type of injury. We observed that grade I injury was characterized by the presence of clustered endothelial cells (CD34-positive cells, purple) distributed along the edge of the clot (figure 1A–C), whereas VWI grade II (figure 1D) and grade III (figure 1E–G) were represented by focal lesions with clusters of endothelial cells, smooth muscle cells (SMA-positive cells, purple), and collagen (light blue on MSB staining, arrows) at the periphery of the clot.

In addition, small foci of CD34- and SMA-positive cells were present inside fragments in two other cases, suggesting early organization of the clots (online supplemental figure 1A–C). A pavement-like surface endothelium representing surface endothelialization was also observed in another case. Dissection of the vertebral artery occurred in one patient.

10.1136/neurintsurg-2021-017310.supp1 Supplementary data

CD34 is not a specific marker for endothelial cells and can be also expressed by hematopoietic stem cells. To deal with this problem, we performed immunohistochemistry for vWF, showing that CD34-positive endothelial cells are also vWF-positive (online supplemental figure 1D, E).

We acknowledge that VWI markers could be also present in a chronic thrombus material and may arise due to a vessel pathology, such as a ruptured atherosclerotic plaque. Therefore, we excluded the presence of foam cells, calcium deposits, fibrous caps or recanalization vessels in VWI clots. In addition, the conventional histopathologic evaluation confirmed that all 33 VWI thrombi were fresh thrombi mainly composed of RBCs and fibrin. These findings indicate that the presence of the endothelial cells, smooth muscle cells and collagen within fresh thrombi was caused by the vessel wall tearing associated with the MT procedure.

### Degree of vessel wall damage and MT devices

As shown in table 1, an aspiration catheter was used in 16 cases (48.5%), a stentriever device in 12 cases (36.4%), and a combined approach in five cases (15.2%). Within the stentriever only group, the stentriever device was changed during the procedure in one case. The same aspiration catheter was used during the entire procedure in each case within the aspiration only group. Within the combination group, the technique was changed using the same stentriever/aspiration catheter during the procedure in each case. There was no significant difference between MT technique with regard to presence of VWI (p=0.7) or the degree of injury (p=0.5).

**Table 1:** Distribution of vessel wall injury (VWI) degree of damage based on mechanical thrombectomy device used.

VWI grade	No of patients (%)		
	Aspiration	Stentriever	Combination
Grade I	13 (39.4)	8 (24.2)	3 (9.1)
Grade II	1 (3)	2 (6.1)	–
Grade III	2 (6.1)	2 (6.1)	2 (6.1)

### Thrombolytic treatment

The IV rtPA administration was correlated with a lower rate of VWI. The rate of VWI was 8% (6/80 cases) for the rtPA group and 17% (27/157 cases) for non-rtPA group (χ^2^=3.93, p=0.04*).

### Number of passes and revascularization outcome

**Figure 2:**
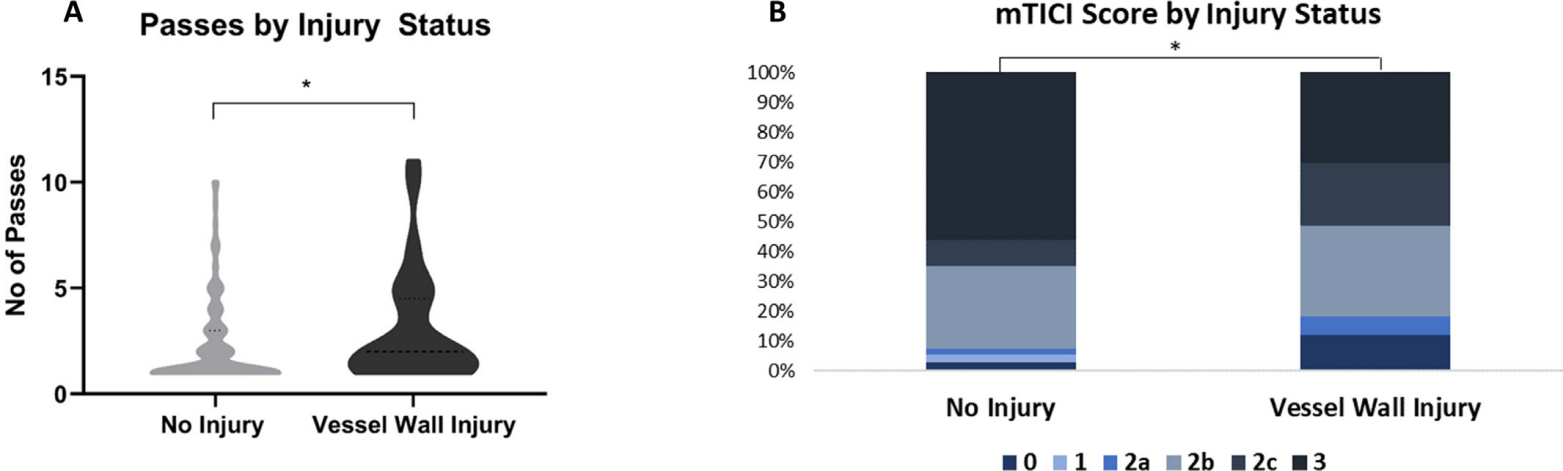
Number of procedural passes and revascularization outcome in cases associated with vessel wall injury (VWI). (A) Violin plots show that patients with VWI had a significantly higher number of passes than non-VWI cases. (B) Graphical representation of final modified Thrombolysis in Cerebral Infarction (mTICI) score for the two groups of patients. VWI was associated with a significantly lower mTICI score in patients with no injury than in patients without VWI.

As represented in figure 2A, the cases associated with VWI (n=33) showed a significantly higher number of procedural passes than non-VWI cases (n=204; two passes (1-4) vs one pass(1-3) H1=4.83, p=0.028*). Moreover, the degree of injury significantly increased with the number of passes (H2=6.72, p=0.035*).

As a limitation of our study, the clots were not collected per pass in all cases and therefore, we could not appreciate in some cases the exact pass when VWI occurred. Nevertheless, more than 3 passes were required to extract the clots in 11 cases with VWI. In particular, in four cases VWI occurred at pass 4, 5, 7 and 11.

Figure 2B highlights that VWI-positive cases (n=33) had a significantly poorer recanalization outcome than non-VWI patients (n=202; H1=6.62, p=0.01*). In particular, there was a significant difference between bad/successful (mTICI ≤2c) and complete revascularization (mTICI 3) outcome for the incidence of VWI (21%, 23/112 cases vs 9%, 10/123 cases, χ^2^=6.48, p=0.01*).

Clinical and procedural information for the two groups of patients are summarized in table 2.

**Table 2:** Clinical characteristics of patients grouped by vessel wall injury status

Characteristic	No of patients (%)	
	No injury, 204 (86)	Vessel wall injury, 33 (14)
MT device		
Aspiration only	84 (41.2)	16 (48.5)
Stentriever only	89 (43.6)	12 (36.4)
Combination	31 (15.2)	5 (15.2)
Stroke Etiology		
Large artery atherosclerosis	36 (17.6)	7 (21.2)
Cardioembolic	88 (43.1)	12 (36.4)
Other determined etiology	16 (7.8)	3 (9.1)
Cryptogenic	49 (24)	9 (27.3)
Not available	15 (7.4)	2 (6.1)
IV rtPA		
Yes	74 (36.3)	6 (18.2)
No	130 (63.7)	27 (81.8)
Statistical analysis	N=237, H1=3.93, p=0.04*	
Occlusion location		
MCA	145 (71.1)	20 (60.6)
ICA/ICA terminus	26 (12.7)	7 (21.2)
Vertebrobasilar	12 (5.9)	1 (3)
Tandem occlusion	11 (5.4)	5 (15.2)
Other dual occlusion*	1 (0.5)	-
Other single location†	4 (2)	-
Multiple locations‡	2 (1)	-
Not available	3 (1.5)	-
No of passes		
1	118 (57.8)	12 (36.4)
2	32 (15.7)	10 (30.3)
3	19 (9.3)	1 (3)
4	11 (5.4)	2 (6.1)
≥5	24 (11.8)	8 (24.2)
Median (IQ1-IQ3)	1 (1-3)	2 (1-4)
Statistical analysis	N=237, H1=4.83, p=0.028*	
Final mTICI score		
0	6 (2.9)	4 (12.1)
1	5 (2.5)	-
2a	4 (2)	2 (6.1)
2b	56 (27.4)	10 (30.3)
2c	18 (8.8)	7 (21.2)
3	113 (55.4)	10 (30.3)
Not available	2 (1)	-
Statistical analysis	N=235, H1=6.62, p=0.01*	

*ICA+PCA; ^†^ACA (one case), PCA (three cases); ^‡^ICA+MCA+ACA.

MT, mechanical thrombectomy; IV rtPA, intravenous recombinant tissue plasminogen activator; MCA, middle cerebral artery; ICA, internal carotid artery; PCA, posterior cerebral artery; ACA, anterior cerebral artery; mTICI Score, modified Thrombolysis in Cerebral Infarction Score.

## Discussion

In this study, we performed a comprehensive histological and immunohistochemical analysis of clots retrieved by MT aimed to identify the presence of vessel wall components, such as endothelial cells, smooth muscle cells, and subendothelial connective tissue which may indicate a VWI caused by MT devices. We found evidence of VWI in 12% of the clots (14% of the cases investigated). The degree of damage varied from grade I (mild intimal damage with CD34-positive cells extended along the edge of the clot), representing the predominant type of injury, to grade II (relevant intimal and subintimal damage), and grade III (severe wall damage) characterized by focal clusters at the margin of the clot containing endothelial cells, smooth muscle cells, and collagen, respectively. There was no significant correlation between VWI and MT technique used, although aspiration was predominantly associated with VWI grade I. IV rtPA administration correlated with a lower rate of VWI. We also found that VWI clots contained significantly more RBCs and less platelets/other compared to non-VWI clots. VWI cases were associated with a significantly higher number of passes and lower mTICI score than the cases without VWI.

VWI has been reported in animal models and may differ based on the MT device used.^1 5 7–11^ The presence of VWI histological markers in AIS emboli suggests that VWI may be caused by the MT device during the intervention procedure. However, it is also possible that they may have arisen due to a vessel pathology, such as a ruptured atherosclerotic plaque. Histological evaluation of thrombi may provide new insights into the complex interaction between the vessel wall, thrombus, and MT devices.

Two previous studies have assessed the occurrence of VWI using histological analysis of retrieved thrombi.^19 20^ In one study, CD34-positive endothelial cells were identified in 11 out of 48 thrombi (23%) removed with a stentriever, but no subendothelial connective tissue was found. Retrieved clots were classified as fibrin dominant (13 clots), RBCs-dominant (15 clots and mixed (20 clots).^19^ In a recent study, in a total of 150 thrombi from 101 patients treated with Penumbra aspiration catheters (42 patients), stentrievers (21 patients), or both techniques (38 patients), collagen fibers and internal elastic lamina were shown in 16% and 8% of specimens, respectively. No difference between aspiration and stentrievers was observed. A low proportion of red blood cells, a high frequency of M2 segment of middle cerebral artery/P2 segment of posterior cerebral artery reached by devices, and a high number of passes were associated with the presence of vascular wall components.^20^ We also found that VWI was associated with a significantly higher number of passes. Moreover, the recanalization outcome was poorer in VWI cases. However, the incidence of VWI in our study was lower and the occlusion was predominantly located in the M1 segment of the middle cerebral artery (64% of VWI cases vs 60% of non-VWI cases). We identified the presence of collagen fibers in six cases (VWI grade III).

Interestingly, in our study, VWI clots contained more RBCs and less platelets/other than non-VWI clots suggesting soft consistency, a finding that argues with the previous study showing that a stiffer clot is associated with higher number of passes and consequently, with VWI. The number of VWI cases in our study is small which may be a factor. On the other hand, Funatsu et al reported that vessel wall components included fibrous caps of plaque. We excluded in our cases the presence of fibrous caps or other plaque components associated with a chronic thrombus material which would indicate a LAA origin rather than a VWI during MT. In our cohort, 7 cases with VWI had LAA etiology. It has been recently reported that LAA clots are richer in RBCs in passes 1-3 than all other suspected etiologies whereas fibrin and platelet/other content increases in clots removed in later passes. 21 We can only assume that VWI in LAA cases may have occurred during early procedural passes.

We also found that IV rtPA administration correlated with a lower rate of VWI. A recent study demonstrated that thrombolysis significantly reduced the clot size,^22^ which may help to retrieve the clot without damaging the occluded vessel. Furthermore, a recent three-dimensional scanning electron microscopy analysis of AIS emboli showed that rt-PA treatment resulted in thinning of fibrin fibers and disruption of fibrin network which may explain the reduced clot size.^23^

A recent study in an animal model reported more intima and medial layer damage after stentriever devices than with aspiration. Several factors may account for this difference, such as the mechanism of clot removal. Aspiration exerts a force to the proximal base of the clot, whereas stentrievers apply a continuous radial force against the vessel wall which may lead to a deeper damage.^1^ Moreover, the radial force varies greatly between stentrievers,^24^ and the extent of vascular injury may increase with the increasing radial force of the stentriever.^10^ Using an in vitro live cell artificial vessel system, Teng *et al* demonstrated that the degree of endothelial injury was related to the vessel diameter in a device-specific manner, with different patterns of endothelial damage caused by different MT devices.^25^ In the present study, VWI grade I was observed in aspiration-retrieved clots more than with a stentriever and was characterized by the presence of endothelial cells extended along the edge of the clot suggesting a large circumferential injury. VWI grades II and III were described more in clots retrieved with stentrievers/combination of devices, showing defined clusters of endothelial cells, smooth muscle cells, and subendothelial connective tissue at the margin of the clot. This pattern may suggest focal endothelial denudation and alteration of tunica media/adventitia.

Our study has several limitations. Chemical and physical manipulation of clots might affect their internal organization and obscure other patterns of injury. MT technique itself may impact clot characteristics. The mTICI scores were self-reported at each site, which might have resulted in some site-to-site variability. Owing to the lack of angiographic data, we were not able to evaluate the occurrence of vasospasm or the presence of an underlying stenosis. Also, a high-resolution MRI scan may provide a better visualization of vessel wall changes. We did not assess in our study other potential factors that could minimize VWI including adjunctive devices such as balloon guiding catheters, anesthesia type, etc. Finally, we included in this study various devices characterized by different radial forces and designs. Further studies could define specific patterns of VWI based on the specific MT device used.

## Conclusions

Histological markers of vascular wall damage were identified in 12% of AIS thrombi. Their presence may indicate that VWI occurred during MT. There was no significant correlation between VWI and the MT device used. Different degrees of damage showed characteristic patterns of injury. VWI was associated with soft thrombus consistency, a higher number of procedural passes and poorer revascularization outcome. Taken together, our findings may improve our understanding of the factors that might affect MT effectiveness in order to achieve fast and complete recanalization without damaging the occluded vessel.

## Data Availability

Data are available from the corresponding author upon reasonable request.
