# Supplementary material for: Histological evaluation of acute ischemic stroke thrombi may indicate the occurrence of vessel wall injury during mechanical thrombectomy
Source: J Neurointerv Surg. 2021 May 11;14(4):356–61. doi: 10.1136/neurintsurg-2021-017310 (PMC8581068; doi:10.1136/neurintsurg-2021-017310)
Supplement: Supplementary data [file neurintsurg-2021-017310supp001.pdf]

SUPPLEMENTARY MATERIAL

SUPPLEMENTARY TABLES:

Supplementary Table 1. Histological composition of retrieved thrombi.

	No injury	Vessel wall injury	Kruskal-Wallis test
Red blood cells (%)	41.78 ± 21.32	52.16 ± 16.88	N=237, H1=7.556, p=0.006*
White blood cells (%)	3.27 ± 2.19	3.48 ± 2.57	N=237, H1=0.027, p=0.869
Fibrin (%)	31.18 ± 16.25	29.15 ± 12.83	N=237, H1=0.148, p=0.701
Platelets/other (%)	23.12 ± 17.88	15.17 ± 14.6	N=237, H1=8.054, p=0.005*
Total ECA (mm^2)	62.75 ± 82.52	43.17 ± 36.28	N=228, H1=1.425, p=0.233

Results are expressed as mean±SD. ECA: extracted clot area.

SUPPLEMENTARY FIGURES:

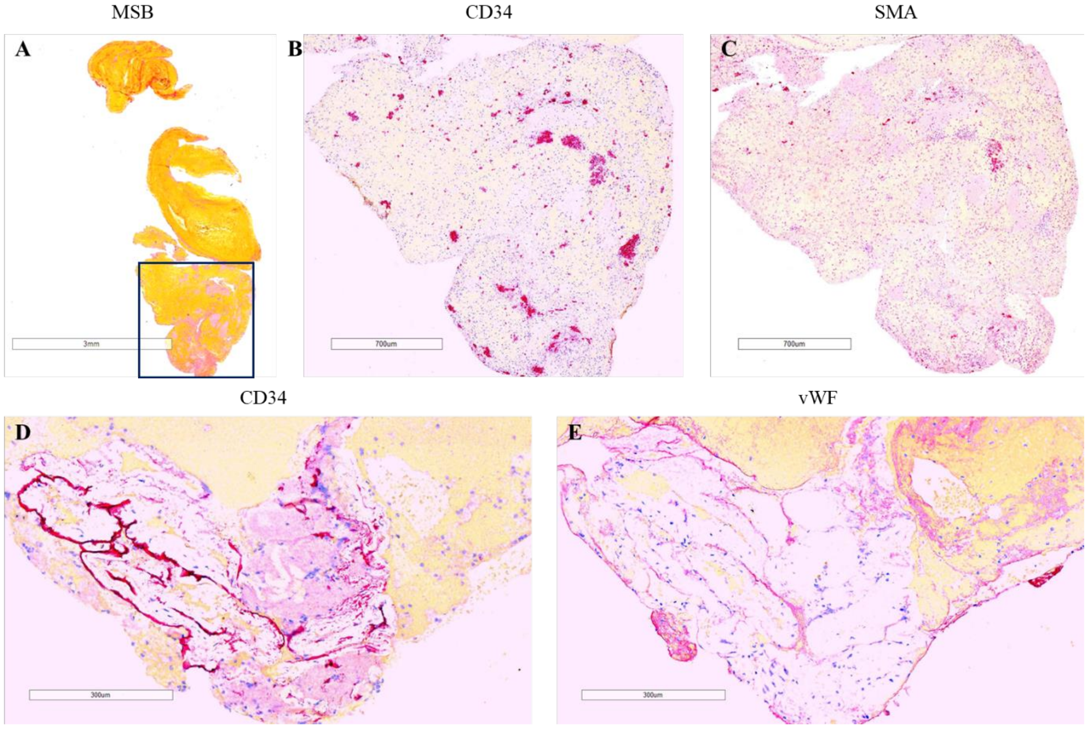

Supplementary Figure 1. Morphological features of thrombi in two representative cases. (A) Martius Scarlett Blue (MSB) staining identifies the main components of clots: red blood cells (yellow), fibrin (red) and platelets/other (light pink). Clot area in the square is showed at higher magnification in the immunostaining images (B, C). Small foci of CD34-positive endothelial cells (B) and smooth muscle actin (SMA)-positive cells (C, purple) were present inside a fragment suggesting early organization of the clot. Immunohistochemistry for CD34 (D) and von Willebrand Factor (E) demonstrates that CD34-positive endothelial cells were also positive for vWF (purple). Scale bar = 3 mm (A), 700 µm (B, C) and 300 µm (D, E).
